# Excess mortality in people hospitalised for alcohol use disorders before and during the pandemic – A registry‐based retrospective cohort study

**DOI:** 10.1111/dar.14045

**Published:** 2025-03-25

**Authors:** Ladislav Kážmér, Ondřej Šíba, Barbora Orlíková

**Affiliations:** ^1^ National Institute of Mental Health Klecany Czech Republic

**Keywords:** alcohol use disorders, COVID pandemic, life expectancy, mortality

## Abstract

**Introduction:**

The aim was to analyse mortality and estimate the life expectancy among people hospitalised for alcohol use disorders (AUD) compared with the general Czech population aged ≥20 years. A temporal perspective on excess mortality was used, covering three recent calendar periods before and during the pandemic.

**Methods:**

Three retrospective cohorts of the target population were constructed using registry‐based data. The target population was defined as all adult patients (aged ≥20 years) admitted to the hospital for AUD (ICD‐10 dg. of F10.x) between 2010 and 2021. Age‐adjusted mortality rates and life expectancies were calculated for the comparative analysis. Official Czech mortality and vital statistics were used for the comparison. A Poisson log‐linear regression model was used to test the effect of the pandemic period (2020–2021) on mortality in the AUD target population.

**Results:**

At age 20, the estimated life expectancy of the AUD target was 21–27 years less than that of the Czech general population. Excess mortality was relatively highest in young people aged 20–34 years and in adults aged 35–49 years. During the pandemic period 2020–2021, mortality rates in the target AUD increased significantly. However, relative inequalities with the general Czech population did not change significantly.

**Discussion and Conclusions:**

People hospitalised for AUD have much higher mortality rates, resulting in markedly reduced life expectancy. During the pandemic, their mortality rates increased even more. However, the increase was no greater than in the general Czech population.

**Key Points**

- People hospitalised for alcohol use disorders (AUD) have a markedly reduced life expectancy.
- In Czechia at age 20, the life expectancy of patients hospitalised for AUD is reduced by 21–27 years.
- During the 2020–2021 period affected by the COVID pandemic, there was an increase in mortality among these AUD patients.

## 1 INTRODUCTION

Alcohol use disorders (AUD) are conditions characterised by compulsive alcohol use and impaired ability to control it, despite their social, economic and health consequences [1]. AUDs encompass a spectrum of such conditions, ranging from mild to severe, and are associated with adverse health outcomes, including excess morbidity and mortality [2, 3].

In the Czech Republic, the prevalence of harmful alcohol use, defined as average daily alcohol intake >60 g for men and >40 g for women, is estimated at 7.3% of the general population aged ≥15 years (10.6% in men and 4.1% in women; data for 2021) [4]. However, research knowledge on excess mortality among people with AUD is limited. One Czech study [5] examined the number and most common causes of death among patients with AUD. The study was rather pilot and descriptive in nature, providing a simple overview of the frequency of deaths and cumulative years of life lost. Another study [6] attempted to estimate the excess risk for all‐cause mortality in people with different types of substance use disorders (SUD) in Czechia, adjusting for a set of physical comorbidities and health conditions. Although specific in its aims, the study did not distinguish between AUD and other SUDs. Instead, it considered the effect of AUD in a pool of all SUDs as a whole rather than looking at AUD separately. The study also pooled all the patients with SUDs over all available time periods since the early 1990s, thus not taking into account temporal changes and possible dynamics in the estimated phenomenon under investigation. Therefore, a more detailed analysis with a specific focus on AUD has not yet been undertaken in Czechia.

The recent COVID‐19 pandemic has affected the lives of people worldwide, including their mental health [7]. The recent review [8] examined how past health and economic crises have affected alcohol consumption. The authors suggested that there could be two opposing outcomes as specific responses to the context of the COVID‐19 pandemic: (i) an increase in alcohol consumption in some populations due to increased psychological distress; or (ii) a decrease in consumption due to reduced physical and financial availability of alcohol during lockdowns [8], as well as reduced opportunities for social drinking due to social distancing measures. As the increased stress and uncertainty associated with the pandemic could have a greater negative impact on vulnerable social groups [9], the first scenario can be particularly relevant for people with high‐risk drinking patterns, including those with AUD. Moreover, a reduction in the accessibility and provision of psychiatric care during lockdowns could have additional adverse effects in this regard. In Czechia, for example, the annual number of hospital admissions for alcohol dependence fell by 15% in 2020–2021, which was a significant drop compared with the temporal trends in previous pre‐pandemic periods [10].

Due to a presumed higher risk of relapse and possible worsening of alcohol problems among high‐risk drinkers, several countries have reported a significant increase in alcohol‐related deaths, such as alcoholic liver disease, during the pandemic period [11, 12, 13]. Some studies have also suggested an association between the presence of SUDs, including alcohol and a higher risk of COVID‐19 infection, as well as severe illness and mortality due to COVID‐19 [14, 15]; although the evidence is not entirely consistent in this regard [16]. Therefore, the pandemic may have both increased mortality in people with AUD and exacerbated existing inequalities in their mortality compared to the general population.

Given this background, the aim of our study was to examine excess mortality and estimate the gap in life expectancy in a population of patients hospitalised for AUD in Czechia. Nationwide, registry‐based data were utilised and designed as a retrospective cohort study. Mortality in AUD patients was analysed in a comparative perspective with the general Czech population, and mortality inequalities between the populations were quantified in a temporal perspective since 2010. The study also focused on the differential impact of the 2020–2021 pandemic period on mortality in AUD patients.

## 2 METHODS

### 2.1 Retrospective cohorts

#### 2.1.1 Target population

The target population was defined as all adult persons aged ≥20 years in Czechia who were in the last 12 years admitted to inpatient psychiatric facilities with AUD as the primary diagnosis (ICD‐10 codes F10.x), starting from 2010 (1 January) to the end of 2021 (31 December). All adult inpatient psychiatric facilities in the country were included; child and adolescent facilities were excluded for formal reasons. Data on admissions were obtained from the National Registry of Reimbursed Health Services (NRRHS), administered by the Institute of Health Information and Statistics of the Czech Republic (IHIS). The NRRHS collects data on all health care providers under public health insurance, which covers almost 100% of the population of the Czech Republic.

#### 2.1.2 Follow‐up

The AUD patients were followed for survival (death event). Survival was tracked by internal linkage to mortality data available in the IHIS's database of death records (DDR) via the patient's unique identification number (ID). The DDR provides records of all deaths in the country that occurred during a calendar period, which are then made available to the National Mortality Registry. The Mortality Registry is administered by the Czech Statistical Office (CzSO) in cooperation with the IHIS.

In the study, we used definitive mortality statistics from the DDR to support the reliability of the provided death counts (as opposed to preliminary ones, where some misreporting could possibly occur). The patient's ID was a pseudonymised code derived from the birth number, both generated and linked internally by the IHIS to the DDR. This internally consistent method was robust to linkage errors that might otherwise be introduced by a misspecified ID.

Follow‐up began on the date of the patient's first hospital admission for AUD in a given calendar period. To analyse temporal changes, the total study period of 12 years was divided into three consecutive periods (*t*): (*t*_1_) 1. 1. 2010–31. 12. 2014; (*t*_2_) 1. 1. 2015–31. 12. 2019 and (*t*_3_) 1. 1. 2020–31. 12. 2021. The *t*_1_, *t*_2_ and *t*_3_ were thus considered as separate cross‐sections. The length of *t*_1_ and *t*_2_ was chosen to provide a sufficient number of follow‐up deaths. To reflect the impact of the COVID pandemic, the length of the last period (*t*_3_) was set at 2 calendar years, as opposed to the previous two periods of 5 years.

Patient survival ended either with the date of death or was censored to the end date of the period *t* (31.12. of the last year of *t*). Exposure time was calculated as the difference between the start of follow‐up and the date of the survival outcome (death or censoring). Both exposure time and survival outcome data, cross‐classified by sex and age group, were generated separately for each of the three calendar periods *t*_*1*_, *t*_*2*_ and *t*_*3*_. Further details on the underlying conceptual framework used to define the follow‐up of the target population can be found in the Appendix, section 1.

### 2.2 Reference population

To quantify the excess mortality in AUD patients, the mortality data for the general Czech population were used as a reference. Annual death statistics and population‐exposed person‐years were obtained from the official sources of the CzSO. The data set, cross‐classified by age group, sex and calendar period, covered the time series from 2010 to 2021 and was pooled into the same three periods (*t*) as the AUD target. To maintain comparability with the AUD, only the population aged ≥20 years was included.

### 2.3 Ethical considerations

The AUD data were pseudonymised and processed internally by the IHIS. The final dataset was provided to the National Institute of Mental Health under a research agreement. Ethical approval for the study was obtained from the Ethical Committee of the National Institute of Mental Health, internal approval number 176/21. The NRRHS registry is a part of the National Health Information System established by §70 par. 1 of Act No. 372/2011 Coll. on health services and conditions of their provision (*Health Services Act*), which eliminates the need for patients' written informed consent. Data on the general Czech population is publicly available from the CzSO's Population and Vital Statistics of the Czech Republic.

### 2.4 Statistical analysis

Several epidemiological techniques were used in the analysis, with data stratified by age (*i*), sex (*j*) and calendar period (*t*). These included the calculation of: (a) mortality rates (crude rates) and age‐specific mortality ratios (MR_i_); (b) directly age‐standardised rates (ASDR) using both external and internal population standards; (c) calculation of life expectancy at a given age (LE_i_); and (d) regression analysis of adjusted mortality rates to formally test for temporal trends in mortality among the AUD target population.

Formal definitions of the rates and ratios referred to in (a) and (b) above are given in Appendix, section 2. For the purpose of data anonymisation and subsequent aggregation, the age at hospital admission of AUD patients was categorised into *k* = 13 age groups (*i*): 20–24, 25–29, …, 75–79, ≥80. For direct ASDR, both external and internal population standards were used, categorised into the same *k* = 13 age groups (external standard – revised European Standard Population [17], internal standard – pooled age structure of AUD target; for definitions see Appendix, section 3).

Estimates of LE_i_ were calculated via the method of abridged life tables, again using the same set of *k* = 13 age groups. To facilitate the presentation of MR_i_, the age groups were pooled into four 15‐year age intervals: 20–34, 35–49, 50–64 and ≥65 years. To examine excess cause‐specific mortality due to COVID‐19 in AUD patients, indirectly standardised mortality ratios were constructed.

Poisson log‐linear regression models were used to test for temporal trends in mortality among AUD patients. The significance of the temporal trends was tested on the dataset of all patients hospitalised for AUD rather than on sex‐specific data. This combined perspective across sexes was used to address issues of statistical power that may be present in sex‐specific data. Poisson regression models were multiple adjusted for age, sex and interaction terms and were fitted to the aggregated AUD dataset as defined in the Appendix, sections 6 to 9. Poisson regression is a widely used approach in epidemiology for modelling mortality rates [18]. It can also be used to test for temporal trends in mortality rates, particularly in situations where only a limited number of time periods are available, as is the case with the data in this study.

## 3 RESULTS

### 3.1 Characteristics of AUD cohorts

Table 1 presents descriptive characteristics of the target cohorts. Both overall and period‐specific descriptive statistics are provided.

**TABLE 1:** Descriptive characteristics of the target population of patients hospitalised for alcohol use disorders by demographics and calendar period.

	Calendar period (*t*)			Total
	2010–2014	2015–2019	2020–2021	
Number of F10.x‐hospitalised persons				
Men	18,187	17,781	7716	43,684
Women	8078	7979	3456	19,513
20–34^a^	4964	4410	1830	11,204
35–49^a^	11,047	11,209	4933	27,189
50–64^a^	8660	8309	3575	20,544
≥65^a^	1594	1832	834	4260
Total	26,265	25,760	11,172	63,197
Number of deaths				
Men	2023	2004	412	4439
Women	551	505	109	1165
20–34^a^	176	175	36	387
35–49^a^	794	841	185	1820
50–64^a^	1211	1113	213	2537
≥65^a^	393	380	87	860
Total	2574	2509	521	5604
Exposure time, in person‐years (p‐yrs)				
Men, sum of p‐yrs	46,502	45,917	8247	100,666
Women, sum of p‐yrs	21,728	21,320	3819	46,867
Total, sum of p‐yrs	68,230	67,237	12,066	147,533
Total, mean (SD)	2.60 (1.51)	2.61 (1.50)	1.08 (0.59)	2.33 (1.51)
Total, median (IQR)	2.66 (2.70)	2.64 (2.68)	1.13 (1.03)	2.05 (2.66)

*Note*: Each patient was followed up from his/her first hospital admission for F10.x diagnosis during the calendar period *t*. If the outcome event (death) was not observed, the exposure was censored on 31st December of the last year of each calendar period *t*. Given the series of three calendar periods, patients could be followed more than once, if there were repeated admissions across the periods. The maximum number of exposures was therefore three, provided that the AUD patient was hospitalised in all three periods *t*_1_ through *t*_3_.

Abbreviations: IQR, interquartile range; SD, standard deviation.

^a^ Age of patient at first hospital admission for F10.x during the calendar period *t*.

A total of 63,197 records of people hospitalised for AUD were followed up altogether in three time periods. The majority were men (43,684; 69%) compared with women (19,513; 31%). Similar proportions were also found for exposure time – the number of person‐years summed over the periods: men 100,666 (68%); women 46,867 (32%). However, for the number of deaths, the predominance of men was more pronounced (men deaths: 4439; 79%).

When compared with the general Czech population, the target AUD patients were characterised by a markedly younger age structure, unimodally distributed around the middle adult age groups (see Appendix, section 4) – approximately 2/3 of the exposed person‐years in the target AUD population were between the ages of 35 and 59 years (Appendix, section 4).

### 3.2 Men with AUD

Table 2 provides a comparative overview of mortality among men AUD patients aged ≥20 years and the reference Czech men general population.

**TABLE 2:** Mortality among male patients hospitalised for alcohol use disorders (AUD) compared with the general population, age ≥20 years, Czechia, 2010 to 2021.

Men	Calendar period		
	2010–2014	2015–2019	2020–2021
(A) Crude mortality rate, per 1000 person‐years, CDR (95% CI)			
AUD patients	43.5 (41.6–45.4)	43.6 (41.8–45.6)	50.0 (45.2–55.0)
General Czech population, age ≥20	13.2 (13.1–13.2)	13.6 (13.5–13.6)	17.0 (16.9–17.1)
(B) Age‐standardised mortality rates, per 100,000 person‐years, ASDR (95% CI)			
External standard (European standard population, 2013)			
AUD patients	7758 (6807–8708)	7997 (6673–9322)	7412 (5282–9542)
General Czech population, age ≥20	1931 (1924–1939)	1826 (1819–1833)	2144 (2132–2156)
Internal standard (pooled AUD age structure, 2010–2021)			
AUD patients	4436 (4242–4629)	4340 (4150–4530)	4816 (4348–5284)
General Czech population, age ≥20	620 (617–624)	550 (547–554)	621 (616–627)
(C) Standardised rate ratios, SRR (95% CI)			
External standard	4.0 (3.6–4.5)	4.4 (3.7–5.2)	3.5 (2.6–4.6)
Internal standard	7.1 (6.8–7.5)	7.9 (7.5–8.2)	7.7 (7.0–8.5)
(D) Age‐specific mortality ratios,^a^ MR_i_ (95% CI)			
20–34	17.2 (14.5–20.4)	22.3 (18.8–26.2)	26.3 (17.7–37.7)
35–49	13.9 (12.8–15.0)	16.9 (15.7–18.3)	16.9 (14.3–20.0)
50–64	5.6 (5.3–6.0)	6.3 (5.9–6.7)	6.1 (5.2–7.1)
≥65	2.6 (2.4–2.9)	2.2 (1.9–2.5)	2.2 (1.7–2.7)
(E) Estimated life expectancy, LE_i_			
At age 20			
AUD patients (95% CI)	30.5 (29.5–31.5)	29.2 (28.0–30.4)	29.5 (27.7–31.3)
General Czech population	55.7	56.6	55.0
*Difference in LE*_*i*_	*−25.2*	*−27.4*	*−25.6*
At age 30			
AUD patients (95% CI)	23.4 (22.8–24.0)	22.9 (22.3–23.6)	20.7 (19.2–22.2)
General Czech population	46.1	47.0	45.3
*Difference in LE*_*i*_	*−22.7*	*−24.1*	*−24.7*
(F) Cause‐specific mortality on COVID‐19			
Crude mortality rate, per 100,000 person‐years, CDR (number of deaths)			
AUD patients, age ≥20	‐	‐	194.0 (16)
General Czech population, age ≥20	‐	‐	247.7 (20,318)
Standardised mortality ratio, SMR (95% CI)	‐	‐	2.44 (1.39–3.96)

Abbreviations: ASDR, age‐standardised rates; CDR, crude mortality rates; CI, confidence interval; SMR, standardised mortality ratio; SRR, standardised rate ratios.

^a^ For more data on age‐specific mortality rates (M_i_) and ratios (MR_i_), see Appendix, section 5.

Crude mortality rates (CDR) in men with AUD ranged from 43.5 to 50.0 deaths per 1000 person‐years (Table 2, section A). Despite their younger age structure, these unadjusted rates were approximately 3 times higher than in the Czech men reference population. From a temporal view, the COVID *t*_3_ calendar period was characterised by increased CDRs (Table 2, section A).

The comparative analysis of ASDR was sensitive to the population standard used (Table 2, sections B and C). When the external European standard was considered, the ASDRs in the men AUD target ranged from 7412 to 7997 per 100,000 person‐years, which were 3.5 to 4.4 times higher rates than in the general men population. When the internal standard was used, the inequalities were even greater, ranging from 7.1 to 7.9 times higher rates in the AUD target, as documented by the corresponding standardised rate ratios (Table 2, section C). When looking at time trends, we refer to the internally standardised ASDRs for AUD men, as these better reflect their age structure. Specifically, similar ASDRs of 4436 in *t*_1_ and 4340 in *t*_2_ were followed by a notable increase to 4816 in *t*_3_ (Table 2, section B).

MR_i_ documents the magnitude of inequalities in mortality between the target and reference populations as a function of age (Table 2, section D). The largest inequalities were found in the relatively young age groups 20–34 and 35–49 (17.2 to 26.3 times higher rates for AUD men; 13.9 to 16.9, respectively). The relatively smallest inequalities were found among those aged ≥65 years (MR_≥65_ ranging from 2.2 to 2.6). From a temporal perspective, the MR_i_ increased significantly only in the first two calendar periods, particularly in the age group 35–49 (Table 2, section D): an increase from 13.9 (95% confidence interval [CI] 12.76–15.03) in *t*_1_ to 16.9 (95% CI 15.65–18.26) in *t*_2_. In contrast, the temporal changes in MR_i_ in the *t*_3_ COVID period were not statistically significant (Table 2, section D).

There was a large loss in estimated life expectancy among men with AUD compared with the reference population (Table 2, section E). At the age of 20, the life expectancy of men with AUD was 25.2 to 27.4 years less than that of Czech men of the same age. At the age of 30, the estimated loss ranged from 22.7 to 24.7 years.

Regarding the cause‐specific deaths of COVID‐19 (Table 2, section F) – although there were only 16 men with AUD died due to COVID‐19 as the underlying cause of death in the calendar period 2020–2021, the mortality due to COVID‐19 among this group was found to be significantly higher than that of the reference Czech men: standardised mortality ratios (95% CI) = 2.44 (1.39–3.96).

### 3.3 Women with AUD

Table 3 presents a comparative analysis of mortality among women with AUD aged ≥20 years and the reference Czech women general population.

**TABLE 3:** Mortality among women patients hospitalised for alcohol use disorders (AUD) compared with the general population, age ≥20 years, Czechia, 2010 to 2021.

Women	Calendar period		
	2010–2014	2015–2019	2020–2021
(A) Crude mortality rate, per 1000 person‐years, CDR (95% CI)			
AUD patients	25.4 (23.3–27.6)	23.7 (21.7–25.8)	28.5 (23.4–34.4)
General Czech population, age ≥20	12.2 (12.1–12.2)	12.5 (12.5–12.6)	14.9 (14.9–15.0)
(B) Age‐standardised mortality rates, per 100,000 person‐years, ASDR (95% CI)			
External standard (European standard population, 2013)			
AUD patients	3897 (2999–4795)	3508 (2701–4316)	3511 (2185–4837)
General Czech population, age ≥20	1263 (1258–1268)	1214 (1210–1219)	1363 (1356–1371)
Internal standard (pooled AUD age structure, 2010–2021)			
AUD patients	2604 (2385–2822)	2337 (2133–2541)	2746 (2226–3267)
General Czech population, age ≥20	336 (334–339)	308 (305–310)	345 (341–349)
(C) Standardised rate ratios, SRR (95% CI)			
External standard	3.1 (2.5–3.9)	2.9 (2.3–3.6)	2.6 (1.8–3.8)
Internal standard	7.7 (7.1–8.4)	7.6 (7.0–8.3)	8.0 (6.6–9.6)
(D) Age‐specific mortality ratios,^a^ MR_i_ (95% CI)			
20–34	34.7 (24.4–48.1)	25.3 (16.2–37.8)	31.9 (11.7–70.0)
35–49	18.5 (15.9–21.5)	16.3 (13.7–19.2)	21.3 (15.2–29.0)
50–64	6.6 (5.8–7.4)	7.0 (6.1–8.0)	7.0 (5.1–9.3)
≥65	1.4 (1.1–1.8)	1.5 (1.2–1.9)	1.1 (0.7–1.8)
(E) Estimated life expectancy, LE_i_			
At age 20			
AUD patients (95% CI)	37.6 (35.6–39.7)	41.0 (39.2–42.7)	38.0 (33.6–42.5)
General Czech population	61.9	62.4	61.1
*Difference in LE*_*i*_	*−24.3*	*−21.4*	*−23.1*
At age 30			
AUD patients (95% CI)	30.7 (29.2–32.1)	32.2 (30.8–33.7)	30.2 (26.6–33.8)
General Czech population	52.0	52.6	51.3
*Difference in LE*_*i*_	*−21.4*	*−20.3*	*−21.1*
(F) Cause‐specific mortality on COVID‐19			
Crude mortality rate, per 100,000 person‐years, CDR (number of deaths)			
AUD patients, age ≥20	‐	‐	0.0 (0)
General Czech population, age ≥20	‐	‐	177.0 (15,250)
Standardised mortality ratio, SMR (95% CI)	‐	‐	Not applicable

Abbreviations: ASDR, age‐standardised rates; CDR, crude mortality rates; CI, confidence interval; SMR, standardised mortality ratio; SRR, standardised rate ratios.

^a^ For more data on age‐specific mortality rates (M_i_) and ratios (MR_i_), see Appendix, section 5.

In women with AUD, the CDR ranged from 23.7 to 28.5 deaths per 1000 person‐years (Table 3, section A). The CDRs were approximately twice as high as those in women from the reference population.

Similar to men, the comparative analysis of ASDR in women was sensitive to the population standard used (Table 3, sections B and C). While for the external standard, the ASDRs in AUD women ranged from 3508 to 3897 per 100,000 person‐years, the internally standardised ASDRs were lower (from 2337 to 2746 per 100,000 person‐years). From a temporal perspective, the internally standardised ASDRs first indicated a notable decrease from 2604 in *t*_*1*_ to 2337 in *t*_*2*_, followed by an increase to 2746 in *t*_*3*_ (Table 3, section B). In terms of relative inequalities, the internally standardised rate ratios indicated approximately eightfold (7.6 to 8.0) higher mortality for women hospitalised for AUD (Table 3, section C).

The excess mortality in AUD women was differentiated by age (Table 3, section D). At the same time, the magnitude of the excess tended to be larger in women than in men, especially in the young age group. Specifically, the MR_20–34_ in women aged 20–34 years ranged from 25.3 to 34.7 (Table 3, section D). Similarly, the MR_35–49_ ranged from 16.3 to 21.3. In contrast, the MR_≥65_ in women aged ≥65 years varied only from 1.1 to 1.5 (Table 3, section D).

From a temporal perspective, the MR_i_ in women did not change significantly (Table 3, section D). Although in the *t*_3_ COVID period, there was an increase in both MR_20–34_ and MR_35–49_ compared with the previous *t*_2_, the increase was not significant: for the pooled 20–49 age group, the Mantel–Haenszel test of homogeneity of risk ratios between *t*_3_ and *t*_2_: *χ* ^2^(1) = 2.18, *p*‐value = 0.140.

Estimated life expectancy for women with AUD showed a large loss of life years (Table 3, section E). At age 20, the estimated loss was 21.4 to 24.3 years, and at age 30, 20.3 to 21.4 years.

In terms of cause‐specific mortality, there were no women with AUD died due to COVID‐19 as the underlying cause of death in 2020–2021 (Table 3, section F), making the comparison with the general Czech women impossible.

### 3.4 Temporal trends and the pandemic period (men and women with AUD combined)

The significance of temporal trends was tested on the dataset of all patients hospitalised for AUD, rather than on sex‐specific data. Nevertheless, testing for temporal trends in all AUD target patients essentially confirmed the descriptive results of the internally standardised ASDRs previously reported in subsections 3.2 and 3.3 from a sex‐specific perspective.

As shown in Table 4, the adjusted estimate of 1.13 times (95% CI 1.026–1.239) higher mortality rate in the pandemic period *t*_3_ compared to the preceding *t*_2_ was proved to be significant.

**TABLE 4:** Testing for temporal trends in mortality among the target patients hospitalised for alcohol use disorders (men and women combined). Outputs from multiple Poisson log‐linear regressions.

Calendar period	Coef.^a^	*p*‐value	Exp (Coef.)^b^
	(S.E.)		(95% CI)
2010–2014^c^	0.015	0.634	1.015
	(0.031)		(0.955–1.079)
2015–2019	Reference period		
2020–2021	0.120*	0.013	1.127*
	(0.048)		(1.026–1.239)
Model descriptive statistics			
Number of observations *N* = 78; *d.f*. = 70			
Pseudo *R* ^2^ = 0.828			
Goodness of fit statistics			
Deviance = 72.28; *p* > *χ* ^2^ (70) = 0.403			
Pearson *χ* ^2^ = 73.78; *p* > *χ* ^2^ (70) = 0.356			

*Note*: The number of observations (78) = 13 age groups × 2 sexes x 3 calendar periods.

Abbreviation: CI, confidence interval.

^a^ Regression coefficients are multiple adjusted for the effects of sex, age groups and interaction terms (*p* = 8 parameters including the intercept). See the Appendix for the full definition of the model (Appendix, section 7.A).

^b^ The exponentiated regression coefficients are rate ratios, that is ratios comparing adjusted mortality rates between two calendar periods.

^c^ In the regression model, interaction terms with the period of 2010–2014 were identified. Here, the regression coefficient for 2010–2014 refers to an estimated effect of the period for men aged 50–54 years.

* *p* < 0.05.

## 4 DISCUSSION

The study provided a complex picture of inequalities in mortality between the target population of people hospitalised for AUD and the corresponding Czech general population. Both period‐specific and temporal perspectives on inequalities were considered, stratified by sex and population age groups.

People hospitalised for AUD have markedly higher mortality rates than the Czech reference population. Depending on the population standard used, the ASDRs for these AUD patients were 4 to 7 times higher for men and 3 to 7 times higher for women. However, excess mortality in AUD patients varied considerably by age. It was highest among young adults aged 20–34 years, followed by those aged 35–49 years.

The excess mortality resulted in a substantial reduction in estimated LE_i_. At age 20, it was estimated to be 25.2 to 27.4 years less for men and 21.4 to 24.3 years less for women. During the pandemic, mortality rates among AUD patients increased significantly, but the inequalities with the Czech reference population did not change, as the increase was no greater than that in the general population.

In developed countries, mortality among people with harmful alcohol use is estimated to be 3–4 times higher than in the general population [19]. Specifically, the UK study [20] suggests that AUD reduces LE_i_ by 17.1 years in men and 10.8 years in women; the Nordic countries [21] report a reduction of 24 to 28 years compared with the general population. Compared with the results of our study, the reduction in LE_i_ in the Czech AUD patients was similar to that in the latter study. At the same time, the formal definition of the target AUD patients used in the Nordic study was closer to that used in our study (patients hospitalised for dg. F10). For formal reasons, it should also be noted that the LE_i_ of the general population in the Nordic countries is 2–4 years higher than in Czechia.

Interestingly, the sex differences in excess mortality among people with AUD appear to vary between European countries. While in Germany [22] a higher relative risk of all‐cause mortality for women was reported, in the UK [20] a higher relative risk was found for men. Nevertheless, the meta‐analysis of data from 81 observational studies across Europe and North America found a significantly higher relative risk of all‐cause mortality in women with AUD than in men with AUD [19]. The study [19] also highlighted that among all people with AUD, the magnitude of the relative risk is much greater in younger age groups than in older ones. Our results fit well with these more general findings. First, we found markedly different relative risks between the age groups, with the highest ones in young AUD patients aged 25–34 years. Second, the relative risks were slightly higher for women than for men, especially at younger ages.

In terms of the containment measures against COVID‐19, several European countries reported higher rates of alcohol consumption in the early stages of the pandemic – the United Kingdom [23], Poland [24] and Germany [25]. The distressing psychosocial factors of the pandemic may have contributed to relapse in people with AUD [26]. People with AUD living alone were particularly at risk of relapse due to COVID‐19‐related factors, such as isolation, lack of family support and psychological distress [27]. In addition, admissions to rehabilitation and addiction centres were severely affected during the pandemic [28], resulting in difficulties in accessing help for alcohol dependence. The research therefore suggests that pre‐pandemic high‐risk drinkers were the most likely to increase their alcohol consumption during the pandemic, compared with social drinkers [8].

In Czechia, the trend during the pandemic appeared to be in a similar direction, with an increase in alcohol use among frequent and high‐risk drinkers, in contrast to a decrease among less frequent drinkers [29]. The availability of psychiatric care for people with AUD was also negatively affected in Czechia. Specifically, in terms of capacity for inpatient care, there was a significant decrease in the annual number of hospital admissions for AUD during the pandemic period [10, 30]. Outpatient care and broader social services for people with AUD were also affected, although to a lesser extent than hospital admissions [10, 30, 31].

The results of our study thus support previous findings on the negative effects of the pandemic from the perspective of the epidemiology of AUDs. First, we found a significant increase in mortality among AUD patients in 2020–2021, over and above the trend observed in previous calendar periods. (Note: As shown in Table 4, the increase in 2020–2021 was found to be statistically significant in the dataset of all patients hospitalised for AUD, combined across sexes, rather than in the sex‐specific approach presented in Tables 2 and 3.) Second, the mortality due to COVID‐19 as the underlying cause of death was also significantly higher in AUD male patients compared with the general male population. Although the number of COVID‐19 deaths in men was too small to provide a robust statistic, the findings suggest that people with AUD were at excess risk in this respect.

### 4.1 Strengths and limitations

The main strength lies in the nationwide, registry‐based data used in the study, which allows for coverage of the entire population, both in terms of the patients hospitalised for AUD and the general population used as a reference. The high‐quality registry data were also derived from a single national population and were therefore collected, processed and analysed in a common framework that does not introduce additional contextual biases that may be present in cross‐national comparative studies.

There are, however, also some limitations that should be kept in mind when interpreting the results of our study. First, the use of inpatient data did not include individuals who received only outpatient care. This omission may therefore imply some selection characteristics of the AUD target examined in the study, for example in terms of relatively higher severity of illness compared to people in outpatient care. Second, the prevalence of AUDs is considered underestimated in the population. Given the presumed underestimation, we can only speculate whether the excess mortality rates and markedly reduced LE_i_ presented in this study are either under‐ or overestimated compared to the ‘full set’ of the AUD population. Third, we did not consider the presence of somatic diseases and possible comorbidities in our study. Similarly, hospital admissions for alcohol‐induced somatic diseases (e.g. alcoholic liver disease, alcoholic gastritis and alcoholic pancreatitis.) – where excess mortality could be even higher – were not explicitly included in the definition of the target AUD patients. All these aspects could therefore be further investigated as additional factors for excess mortality in AUD patients.

Furthermore, regarding the impact of the pandemic period tested in the study, it should be kept in mind that closures of outpatient care during lockdowns could lead to some additional selection effects in the inpatient AUD target. In fact, these could act in opposite directions: (a) an increased need for underserved care could potentially lead to a preference for hospitalisation of the ‘most severe AUD cases’ over ‘less acute’ AUD patients; (b) the pandemic period could also lead to situations in which the most severe AUD patients were unable to access inpatient care during the lockdown period (e.g. if a severe alcohol relapse and/or a fatal COVID‐related illness prevented access to psychiatric care during the period). In case (b), the 2020–2021 AUD cohort would be characterised by a slightly reduced severity compared to the previous periods.

## 5 CONCLUSIONS

In Czechia, mortality among patients hospitalised for AUD was several times higher than in the general population. In 2010–2021, the estimated reduction in life expectancy at age 20 varied between 21 and 27 years for AUD patients. The pandemic period of 2020–2021 had a negative effect in this respect, with an additional increase in mortality rates. However, this increase did not significantly change the magnitude of inequalities between AUD patients and the general Czech population.

Regarding COVID‐19 as the underlying cause of death, mortality among men AUD patients was significantly higher than in the Czech men reference population. The number of COVID‐19 deaths was, however, limited to provide a more robust statistic in this regard.

## AUTHOR CONTRIBUTIONS

LK conceptualised the study design, was responsible for the literature review, performed the analyses, interpreted the results, drafted and revised the manuscript. OŠ and BO participated in the literature review, drafting and revision of the manuscript. Each author certifies that their contribution to this work meets the standards of the International Committee of Medical Journal Editors.

## FUNDING INFORMATION

This study was supported by the Czech Health Research Council, grant number NU22‐D‐146: ‘The impact of the COVID‐19 pandemic on natural causes of death, suicides and self‐harm in individuals with a history of mental disorders: a retrospective cohort study based on nationwide health registers’. The study was also financially supported by the Ministry of Education, Youth and Sports of the Czech Republic, the Operational Programme Johannes Amos Comenius project identification number CZ.02.01.01/00/22_008/0004583: ‘Research of Excellence on Digital Technologies and Wellbeing (DigiWELL)’. The funders had no role in the design, data collection, data analysis and reporting of this study.

## CONFLICT OF INTEREST STATEMENT

The authors have no conflicts of interest to declare.

## Supporting information


**Data S1.** Supporting Information.

## Data Availability

The data that supports the findings of this study are available in the supplementary material of this article.
