# Supplementary material for: Excess mortality in people hospitalised for alcohol use disorders before and during the pandemic – A registry‐based retrospective cohort study
Source: Drug Alcohol Rev. 2025 Mar 25;44(4):1229–39. doi: 10.1111/dar.14045 (PMC12117291; doi:10.1111/dar.14045)
Supplement: Supplementary file 1 — Data S1. Supporting Information. [file DAR-44-1229-s001.pdf]

APPENDIX

List of Appendix sections:

Section 1. The conceptual framework for the follow-up of the target population of the study

Section 2. The definition of adjusted mortality rates and mortality ratios used in the study

Section 3. Population standards used for the computation of directly age-standardised mortality rates (ASDRs)

Section 4. Comparison of the age structure between target patients hospitalised for AUD and the general Czech population, pooled data over t_1 through t_3 (2010–2021), by sex

Section 5. Comparison of age-specific mortality rates between target patients hospitalised for AUD and the general Czech population, by major age groups, sex and calendar period

Section 6. Definition of the Poisson log-linear model

Section 7. Testing for temporal trends in mortality among the target patients hospitalised for AUD (men and women combined). Full definition of the Poisson log-linear model fitted to the aggregated dataset of the target AUD patients

Section 7.A. Model M1 – calendar periods t_1 through t_3 treated as separate cross-sections

Section 7.B. Model M2 – the effect of the pandemic period (t_3) controlled for a broader time trend in the data

Section 7.C. Model descriptive statistics, for both M1 and M2

Section 8. Model-predicted mortality rates as implied by M1 defined in the Appendix, section 7.A. Poisson log-linear regression, target patients hospitalised for AUD, by sex and calendar period

Section 9. The aggregated input dataset of target population used in the study – patients hospitalised for alcohol use disorders (dg. F10.x), Czechia, by sex, age group and calendar period

APPENDIX

1.) The conceptual framework for the follow-up of the target population of the study

In Figure, the underlying framework for the follow-up of the target population of patients hospitalised for alcohol use disorders (AUD) is presented graphically. The framework was used for the initial data extraction from the Czech National Registry of Reimbursed Health Services (NRRHS).

In the framework, the three calendar periods (t_1 through t_3) covered by the study were considered as separate cross-sections, thus providing the follow-up data for three consecutive periods. Considering calendar periods as separate cross-sections is basically an analogous approach to the construction of period life tables, where data are available by *ad-hoc* defined periods (either by a single calendar year or by a group of years).s

Figure. Conceptual framework for follow-up

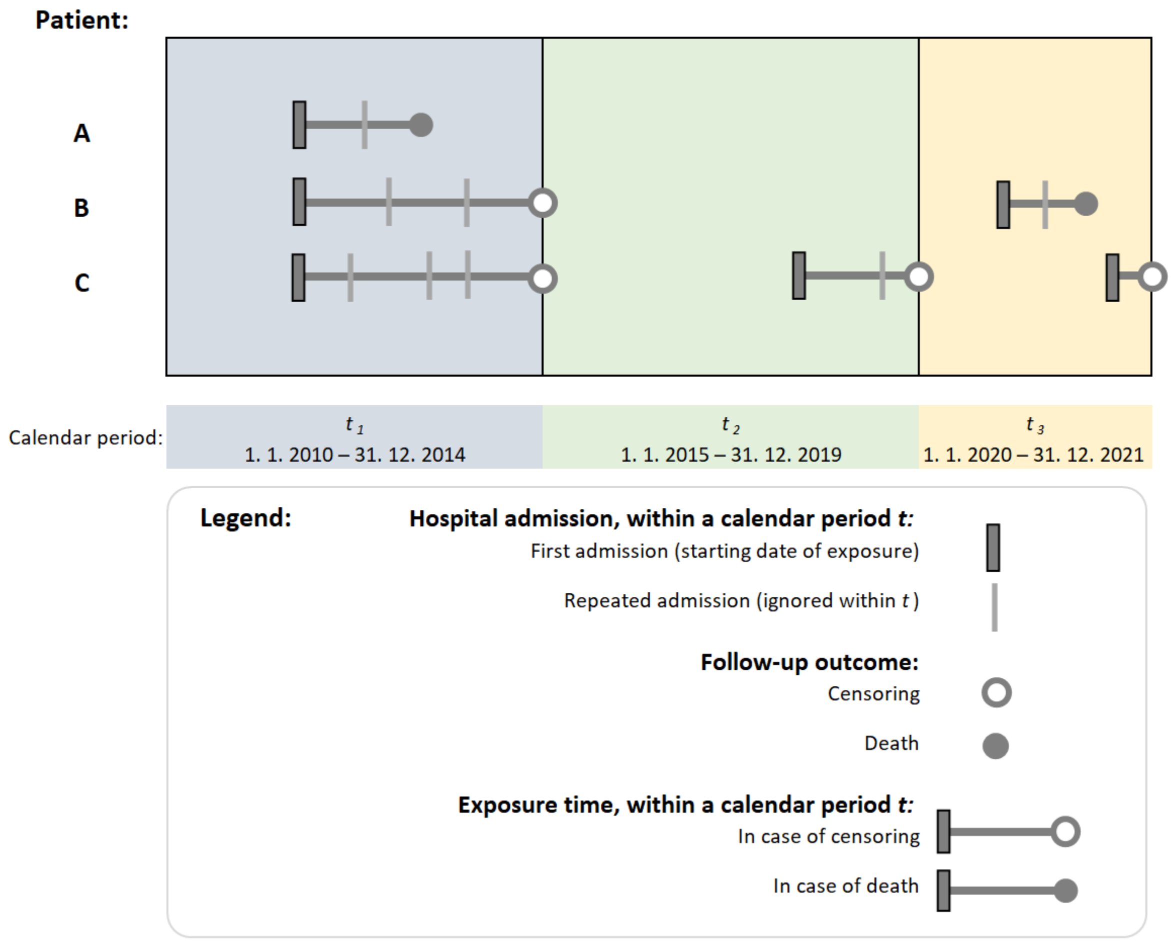

As shown in the Figure, patients hospitalised for AUD could be followed-up more than once in the

study, depending on i) whether there were repeated hospital admissions for dg. F10.x; ii) the timing of these readmissions (*across* calendar periods or *within* a calendar period).

If there were readmissions of the AUD patient *within the same* calendar period t (e.g. patient A in the Figure), the date of the first admission within the given t was considered as the starting point of the patient's follow-up. Thus, the follow-up exposure started on the date of the *first* hospital admission *within* the given calendar period t. Repeated admissions *within the same* calendar period t were unimportant and therefore ignored. The follow-up exposure time of a patient either ended with the date of death or was censored to the end date of the given calendar period *ts*. (Example: If the date of first hospital admission of Patient A within the plotted calendar period t_1 was 1. 7. 2011, and the date of death was 1. 7. 2013, the exposure time was 731 days, i.e. 2 years.)

If there were repeated hospital admissions *in different* calendar periods t, the patient was followed-up more than once (e.g. patients B and C). Given the series of three calendar periods t considered in the study (t_1 = 2010–2014, t_2 = 2015–2019, and t_3 = 2020–2021), the number of follow-up exposures could thus range from one to three. Multiple exposures *across* the different calendar periods were considered as separate observations.

Note: In contrast to the framework used for this study, it should be noted that for period life tables it is a rule of thumb that the length of the calendar periods should be balanced rather than unbalanced. Nevertheless, it should be also emphasised that the time length of the calendar periods used for this study was determined with regards to its specific objectives. First, to consider a sufficiently large sum of the exposed person-years in a follow-up that would avoid extremely low number of death cases in most of the demographic subgroups of the target population, as defined by age and sex. For this objective, the time length of 5 years was chosen as the baseline. Second, the calendar period affected by the Covid pandemic should be considered as a unique cross-section and therefore examined separately. For this second objective, the 2-year time length, starting from 1. 1. 2020 and ending 31. 12. 2021, was chosen for the third calendar period (t_3). Although this implies a certain imbalance with respect to the length of the previous calendar periods, the methods of statistical modelling of mortality rates, including the log-linear Poisson model used in the study (Appendix, sections 6–8), provide the methodological tool to deal with an additional uncertainty in the estimated mortality rates resulting from a shorter follow-up for this third calendar period t_3.

2.) The definition of adjusted mortality rates and mortality ratios used in the study

i – age group
j – sex
t – calendar period
${}^{t}\mathrm{D}_i^j$ – number of incident deaths, in the age group i during t, in sex j
${}^{t}\mathrm{P}_i^j$ – number of exposed person-years, in the age group i during t, in sex j
k – number of age groups, here $k = 13$ (from 20–24, 25–29, … , 75–79, 80+)

${}^{t}\mathrm{CDR}^j$ – crude mortality rate, during period t, in sex j (per 1,000 person-years):

$$ {}^{t}\mathrm{CDR}^j = \frac{\sum_i^k {}^{t}\mathrm{D}_i^j}{\sum_i^k {}^{t}\mathrm{P}_i^j} * 1{,}000 $$

${}^{t}\mathrm{M}_i^j$ – age-specific mortality rate, for the age group i during t, in sex j (per 100 thousand person-years):

$$ {}^{t}\mathrm{M}_i^j = \frac{{}^{t}\mathrm{D}_i^j}{{}^{t}\mathrm{P}_i^j} * 100{,}000 $$

${}^{S}\mathrm{w}_i^j$ – share of the age group i on the total standard population S, in sex j

${}^{t}\mathrm{ASDR}^j$ – age-standardised mortality rate, during period t, in sex j (per 100 thousand person-years):

$$ {}^{t}\mathrm{ASDR}^j = \frac{\sum_i^k {}^{S}\mathrm{w}_i^j * {}^{t}\mathrm{M}_i^j}{\sum_i^k {}^{S}\mathrm{w}_i^j} * 100{,}000 $$

${}^{t}\mathrm{SRR}^j$ – standardised rate ratio, during period t, in sex j:

$$ {}^{t}\mathrm{SRR}^j = \frac{{}^{t}\mathrm{ASDR}^j\ (target\ population)}{{}^{t}\mathrm{ASDR}^j\ (reference\ population)} $$

${}^{t}\mathrm{MR}_i^j$ – age-specific mortality ratio, for the age group i during t, in sex j:

$$ {}^{t}\mathrm{MR}_i^j = \frac{{}^{t}\mathrm{M}_i^j (target\ population)}{{}^{t}\mathrm{M}_i^j (reference\ population)} $$

${}^{t}\mathrm{SMR}^j$ – standardised mortality ratio, during t, in sex j:

$$ {}^{t}\mathrm{SMR}^j = \frac{\sum_i^k {}^{t}\mathrm{D}_i^j (target\ population)}{\sum_i^k {}^{t}\mathrm{M}_i^j (reference\ population) * {}^{t}\mathrm{P}_i^j (target\ population)} $$

3.) Population standards used for the computation of directly age-standardised mortality rates (ASDRs)

In this study, the external population standard refers to the revised European Standard Population (2013), with the 5-year age groups pooled into the $k = 13$ categories to maintain formal consistency with the AUD dataset. The internal standard, stratified by sex, was derived directly from the AUD dataset by pooling the observed age structure over all the three calendar periods t_1 through t_3.

i) External standard

European standard population (2013):

Age group (*i*)	^{s}w
20–24	7.6 %
25–29	7.6 %
30–34	8.3 %
35–39	8.9 %
40–44	8.9 %
45–49	8.9 %
50–54	8.9 %
55–59	8.3 %
60–64	7.6 %
65–69	7.0 %
70–74	6.4 %
75–79	5.1 %
≥80	6.4 %
Total	100.0 %

Note: The European standard population has the same age-structure for both sexes.

ii) Internal standard

Age-structure of the target population of AUD patients pooled over t_1 through t_3 (2010–2021), by sex:

Age group (*i*)	Sex (*j*)	
	Men	Women
20–24	2.7%	2.4%
25–29	6.3%	4.6%
30–34	10.6%	8.7%
35–40	14.3%	13.0%
40–44	15.3%	14.8%
45–50	14.5%	14.9%
50–54	13.0%	14.6%
55–59	10.8%	12.4%
60–64	7.2%	8.0%
65–69	3.4%	4.2%
70–74	1.4%	1.7%
75–79	0.4%	0.4%
≥80	0.1%	0.2%
Total	100.0%	100.0%

There are substantial differences in the age structure between the two standards. Compared to the internal standard of AUD target patients (2010–2021), the age structure of the external European standard population (ESP, 2013) is more homogeneous across age groups. Therefore, the weights for older age groups are markedly higher in the ESP (2013) than in the internal standard of AUD patients. These differences are implicitly responsible for the markedly different magnitude of the age-standardised rates (ASDRs) when applying the respective population standards.

4.) Comparison of the age structure between target patients hospitalised for AUD and the general Czech population, pooled data over t_1 through t_3 (2010–2021), by sex

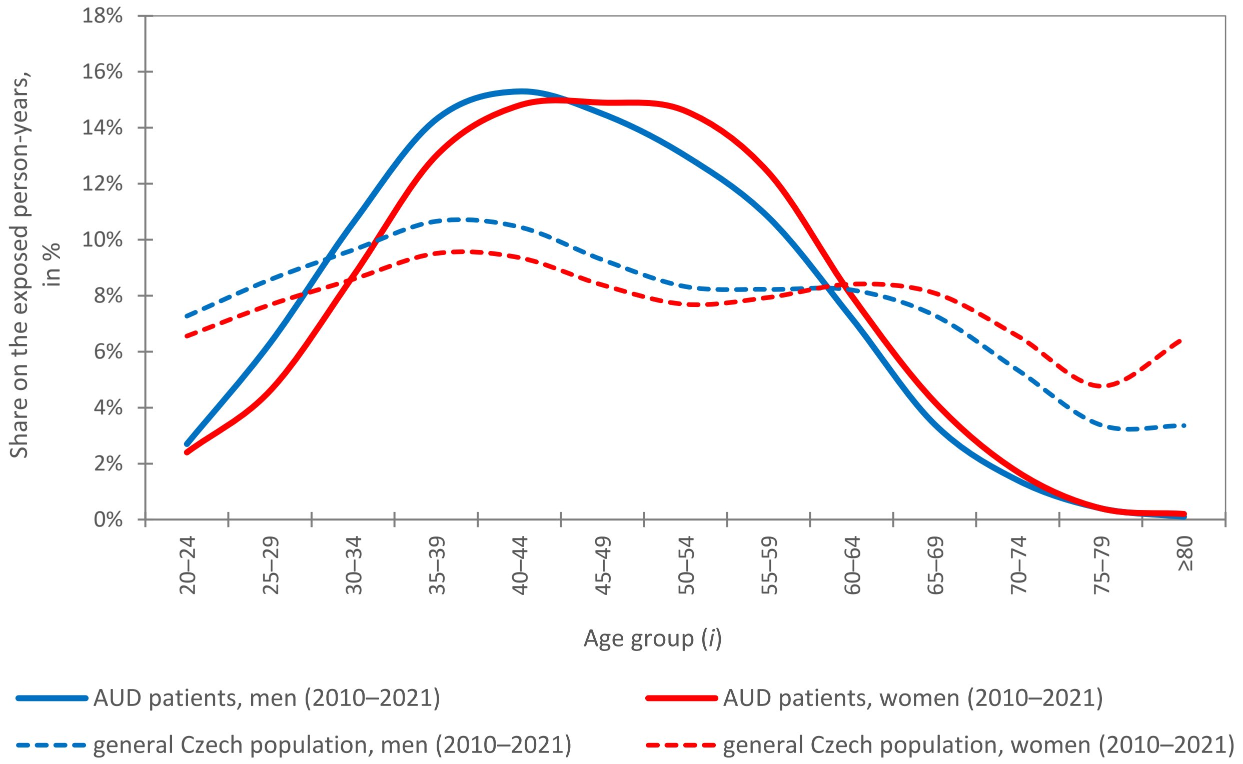

5.) Comparison of age-specific mortality rates between target patients hospitalised for AUD and the general Czech population, by major age groups, sex and calendar period

The following table provides with additional age-specific data on the excess mortality of AUD patients, as compared to their reference general Czech population. The table is provided in order to further document the results presented in Tables 2 and 3 in the main text. For the sake of simplicity, the data are presented in major age groups with an age range of 15 years.

Sex: Men

Age group (*i*)	Period of 2010–2014			Period of 2015–2019			Period of 2020–2021		
	M_i rate (per 1,000 person-years)		MR_i ratio	M_i rate (per 1,000 person-years)		MR_i ratio	M_i rate (per 1,000 person-years)		MR_i ratio
	AUD patients	general Czech population		AUD patients	general Czech population		AUD patients	general Czech population	
20–34	14.3	0.8	17.2	17.7	0.8	22.3	20.6	0.8	26.3
35–49	30.8	2.2	13.9	33.5	2.0	16.9	38.7	2.3	16.9
50–64	65.0	11.6	5.6	62.2	9.9	6.3	66.2	10.8	6.1
≥65	142.7	54.2	2.6	111.6	50.9	2.2	132.6	61.1	2.2
Total (age ≥20 years)	43.5	13.2	3.3	43.6	13.6	3.2	50.0	17.0	2.9

Sex: Women

Age group (*i*)	Period of 2010–2014			Period of 2015–2019			Period of 2020–2021		
	M_i rate (per 1,000 person-years)		MR_i ratio	M_i rate (per 1,000 person-years)		MR_i ratio	M_i rate (per 1,000 person-years)		MR_i ratio
	AUD patients	general Czech population		AUD patients	general Czech population		AUD patients	general Czech population	
20–34	10.4	0.3	34.7	7.4	0.3	25.3	10.9	0.3	31.9
35–49	19.4	1.1	18.5	16.0	1.0	16.3	24.7	1.2	21.3
50–64	33.6	5.1	6.6	32.1	4.6	7.0	34.7	5.0	7.0
≥65	62.7	44.5	1.4	62.9	41.3	1.5	52.6	46.3	1.1
Total (age ≥20 years)	25.4	12.2	2.1	23.7	12.5	1.9	28.5	14.9	1.9

Note: M_i stands for age-specific mortality rate, MR_i for age-specific mortality ratio (i.e. ratio of two rates).

6.) Definition of the Poisson log-linear model

${}^{t}\lambda_i^j$ – parameter of the Poisson distribution (rate); for age group *i*, period *t*, and sex *j*

X – independent variable, predictor or covariate
β – regression coefficient
$\sum_p X_p \beta_p$ – linear combination of *p* predictors, including intercept term

Poisson regression model specification:

$${}^{t}D_i^j \sim Poisson({}^{t}\lambda_i^j)$$

$$\log({}^{t}\lambda_i^j) = \log({}^{t}P_i^j) + \sum_p X_p \beta_p$$

The regression term $\log({}^{t}P_i^j)$ is the population **offset**. It adjusts for unequal exposure to an outcome of interest between the data points. The offset is defined as the log-value of a group-specific exposure ${}^{t}P_i^j$, and has a fixed regression coefficient of 1.

Two equivalent treatments of calendar time on the right-hand side of the regression equation:

To test for temporal trends in mortality among the target patients hospitalised for AUD, two equivalent regression models were constructed, as described further in the Appendix, sections 7.A (Model M1) and 7.B (Model M2).

The rationale for M1 was to test for changes in mortality rates between the three calendar periods considered as separate cross-sections, thus treating them as a set of categorical variables.

In contrast, the rationale for M2 was to estimate the effect of the pandemic period after explicitly adjusting for a broader time trend that may be present in the data. Therefore, a continuous predictor controlling for the time trend was set as the first, and the significance of the additional effect of the pandemic period (2020–2021) was tested as the second. Thus, the effect of the pandemic was tested in terms of deviations of mortality rates in 2020–2021 from those predicted by the controlling predictor.

For the sake of simplicity, Table 4 in the main text of the paper presents only the results from the M1 model definition. As stated in the main text, the Poisson regression models were conducted on the dataset of all target patients hospitalised for AUD (i.e. men and women with AUD combined).

7.) Testing for temporal trends in mortality among the target patients hospitalised for AUD (men and women combined). Full definition of the Poisson log-linear model fitted to the aggregated dataset of the target AUD patients

The number of data points in the aggregated dataset was $N = 78$. The value of N was implied from the following cross-classification of AUD individuals into the grouped data points: $N(78)$ = 13 age groups * 2 sexes * 3 calendar periods

In the Poisson regression model, the age was grouped into $k = 13$ age groups (i): 20–24, 25–29, … , 75–79, ≥80. For each of the age groups i, the midpoint of the 5-year age interval was assigned: 22.5 for i = 20–24, 27.5 for i = 25–29, … , 77.5 for i = 75–79, and 82.5 for the last age group i ≥80. In addition, the age was also centred on the midpoint of the central age group, $i_{central}$ = 50–54 years, i.e. centred on 'age 52.5 years'.

7.A) Model M1 – calendar periods t_1 through t_3 treated as separate cross-sections

(Term)	Predictor	Coef.	(S.E.)	*p*-Value	Exp(Coef.)	Exp(Coef.), (95% CI)
	**Main effects:**					
(a)	Age group (i), centred from i = 50–54	0.045	(0.002)	<0.001	1.046	(1.043–1.050)
	Sex (j), reference j = Men					
(b)	Women	-0.677	(0.045)	<0.001	0.508	(0.465–0.555)
	Period (t), reference t_2 = 2015–2019					
(c)	2010–2014 (t_1)	0.015	(0.031)	0.634	1.015	(0.955–1.079)
(d)	2020–2021 (t_3)	0.120	(0.048)	0.013	1.127	(1.026–1.239)
	**Interaction effects with the Period of 2010–2014:**					
(a) * (c)	Age group (i), centred * Period of 2010–2014 (t_1)	0.012	(0.002)	<0.001	1.012	(1.007–1.017)
(b) * (c)	Women * Period of 2010–2014 (t_1)	0.086	(0.066)	0.195	1.089	(0.957–1.240)
(a) * (b) * (c)	Age group (i), centred * Women * Period of 2010–2014 (t_1)	-0.013	(0.004)	0.001	0.987	(0.978–0.995)
(e)	**Intercept[1]**	-2.980	(0.022)	<0.001	0.051	(0.049–0.053)
(f)	**Offset, Ln(Exposed person-years)**	1.000	.	.	.	.

[1] Central age group $i_{central}$ = 50–54, Sex j = Men, in reference calendar Period t_2 = 2015–2019.
The main effect of term (d) is shaded, i.e. the effect of Period 2020–2021 (t_3).

7.B) Model M2 – the effect of the pandemic period (t_3) controlled for a broader time trend in the data

(Term)	Predictor	Coef.	(S.E.)	*p*-Value	Exp(Coef.)	Exp(Coef.), (95% CI)
	**Main effects:**					
(a)	Age group (*i*), centred from *i* = 50–54	0.045	(0.002)	<0.001	1.046	(1.043–1.050)
	Sex (*j*), reference *j* = Men					
(b)	Women	-0.677	(0.045)	<0.001	0.508	(0.465–0.555)
(c)	Time trend[1], num. of years from the base of 1st January 2010	-0.003	(0.006)	0.634	0.997	(0.985–1.009)
(d)	Period of 2020–2021 (t_3)	0.130	(0.058)	0.024	1.139	(1.017–1.276)
	**Interaction effects with the Period of 2010–2014:**					
(a) * (t_1)	Age group (*i*), centred * Period of 2010–2014 (t_1)	0.012	(0.002)	<0.001	1.012	(1.007–1.017)
(b) * (t_1)	Women * Period of 2010–2014 (t_1)	0.086	(0.066)	0.195	1.089	(0.957–1.240)
(a) * (b) * (t_1)	Age group (*i*), centred * Women * Period of 2010–2014 (t_1)	-0.013	(0.004)	0.001	0.987	(0.978–0.995)
(e)	**Intercept[2]**	-2.957	(0.035)	<0.001	0.052	(0.048–0.056)
(f)	**Offset, Ln(Exposed person-years)**	1.000	.	.	.	.

[1] For the calendar period t_1 (2010–2014) centred on 1st July 2012, the value of the predictor is 2.5 years. Similarly, for the calendar period t_2 (2015–2019) centred on 1st July 2017, the value of the predictor is 7.5 years. For the t_3 (2020–2021), centred on the 31st December 2020, the value of the predictor is 11.0 years.

[2] Central age group $i_{central}$ = 50–54, Sex *j* = Men, on the 1st January 2010 (base of the Time trend)

The main effect of term (d) is shaded, i.e. the effect of Period 2020–2021 (t_3).

The main effect of term (d), the Period of 2020–2021 (t_3), is an adjusted effect of the pandemic period after statistical control for both the time trend and all other controlling predictors included in the regression model.

7.C) Model descriptive statistics, for both M1 and M2

Number of observations	=	78
LR statistic, $\chi^2(d.f.=7)$	=	2,301.09
p-Value > χ^2	=	<0.001
Pseudo R^2	=	0.828
**Goodness of fit statistics:**		
Deviance goodness-of-fit	=	72.28
p-Value > $\chi^2(70)$	=	0.403
Pearson goodness-of-fit	=	73.78
p-Value > $\chi^2(70)$	=	0.356

Goodness of fit statistics test for a consistency between observed and model predicted values of the dependent variable. Non-significant values of the test statistic indicate a good model fit.

8.) Model-predicted mortality rates as implied by M1 defined in the Appendix, section 7.A. Poisson log-linear regression, target patients hospitalised for AUD, by sex and calendar period

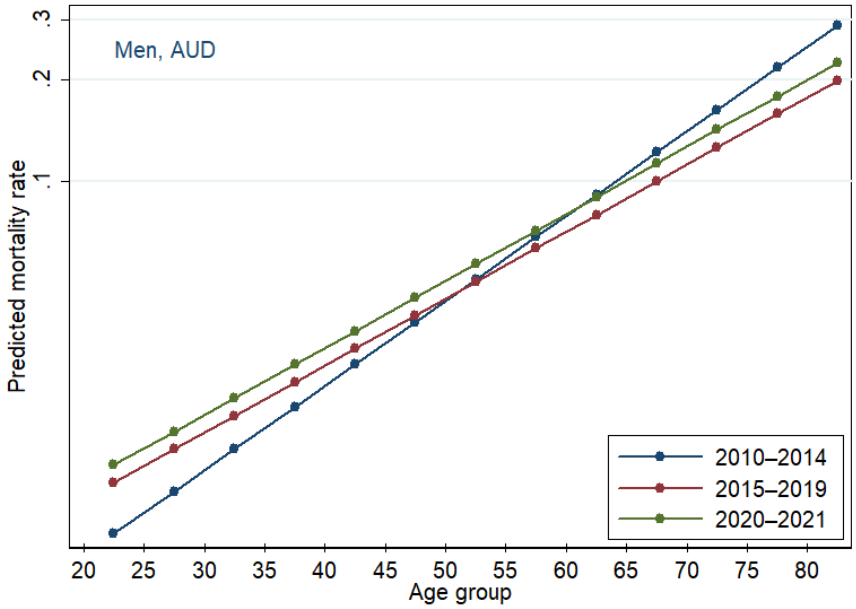

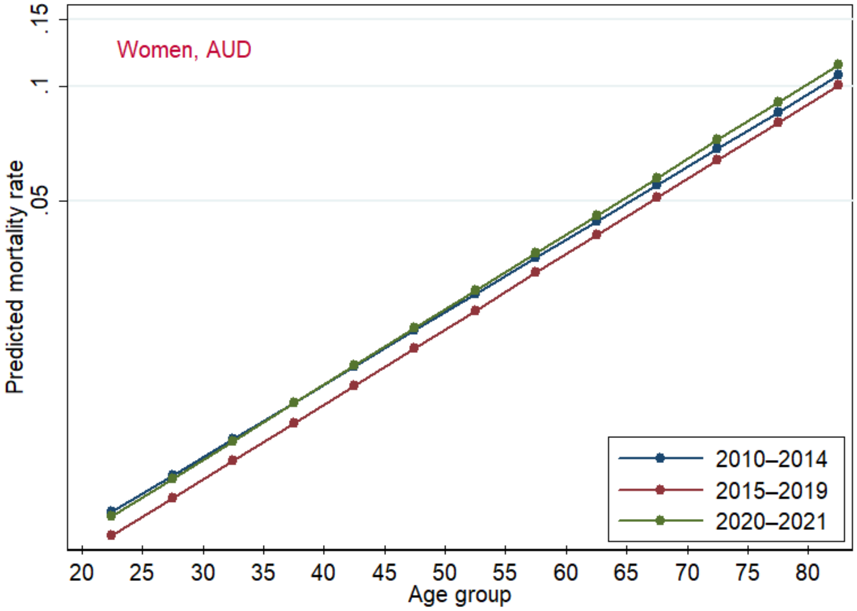

Note: Predicted mortality rates are plotted on a logarithmic scale. For the purpose of data aggregation, the age of AUD patients was grouped into k = 13 age groups (i): 20–24, 25–29, … , 75–79, ≥80. For each of the age groups i, the midpoint of the 5-year age interval was assigned: 22.5 for i = 20–24, 27.5 for i = 25–29, …, 77.5 for i = 75–79, and 82.5 for the last age group i ≥80. In the Poisson regression model, the controlling predictor of age was also centred on the midpoint of the central age group, $i_{central}$ = 50–54 years, i.e. centred on 'age 52.5 years'.

9.) The aggregated input dataset of target population used in the study – patients hospitalised for alcohol use disorders (dg. F10.x), Czechia, by sex, age group and calendar period

Age group (i)	Period of 2010–2014		Period of 2015–2019		Period of 2020–2021	
	Number of deaths (D_i), 2010–2014		Number of deaths (D_i), 2015–2019		Number of deaths (D_i), 2020–2021	
	Men	Women	Men	Women	Men	Women
20–24	14	5	14	1	0	0
25–29	37	9	47	5	5	2
30–34	88	23	90	18	25	4
35–39	138	49	179	44	34	9
40–44	202	61	249	45	49	14
45–49	274	70	267	57	62	17
50–54	301	80	294	85	70	17
55–59	339	103	288	71	55	13
60–64	312	76	294	81	42	16
65–69	172	47	162	56	36	9
70–74	91	15	75	27	21	3
75–79	41	7	23	9	10	4
≥80	14	6	22	6	3	1
Total	2,023	551	2,004	505	412	109

Age group (i)	Period of 2010–2014		Period of 2015–2019		Period of 2020–2021	
	Number of exposed person-years (P_i), 2010–2014		Number of exposed person-years (P_i), 2015–2019		Number of exposed person-years (P_i), 2020–2021	
	Men	Women	Men	Women	Men	Women
20–24	1,447.05	568.07	1,150.10	490.37	145.00	60.32
25–29	3,056.74	1,015.47	2,801.44	1,004.79	457.51	144.27
30–34	5,191.90	1,979.19	4,601.96	1,736.95	850.89	344.33
35–39	6,643.71	2,933.01	6,659.44	2,760.83	1,054.93	415.69
40–44	6,623.08	2,874.97	7,457.76	3,451.03	1,358.77	612.22
45–49	6,655.94	3,463.00	6,626.37	2,937.85	1,330.20	594.11
50–54	6,099.63	3,352.03	5,873.39	2,926.51	1,084.29	561.58
55–59	5,300.20	2,724.55	4,730.66	2,633.64	887.49	434.43
60–64	3,255.06	1,620.90	3,488.59	1,821.34	549.94	328.89
65–69	1,529.09	822.56	1,578.68	968.31	323.47	193.91
70–74	499.79	271.93	738.42	433.39	135.83	88.24
75–79	139.95	60.86	163.72	113.23	57.82	28.84
≥80	60.10	41.30	46.08	42.07	10.97	12.28
Total	46,502.23	21,727.83	45,916.61	21,320.30	8,247.12	3,819.09
